# The limited contribution of DNA methylation to PML-RARα induced leukemia

**DOI:** 10.18632/oncotarget.875

**Published:** 2013-02-15

**Authors:** Christian Rohde, Till Schoofs, Carsten Müller-Tidow

**Affiliations:** Department of Medicine A - Hematology, Oncology and Pneumology, University of Münster, Germany; Department of Medicine A - Hematology, Oncology and Pneumology, University of Münster, Germany; Department of Medicine A - Hematology, Oncology and Pneumology, University of Münster, Germany

Cellular transformation by oncogenes involves disturbance of multiple cellular systems. Epigenetic changes occur frequently in transformed cells and might serve several functions. On the one hand side, epigenetic alterations such as altered promoter DNA methylation could contribute to the malignant phenotype by affecting gene expression levels. On an even more global level, epigenetic marks and especially DNA methylation could corroborate the malignant phenotype by disrupting the three dimensional chromosomal architecture.

A variety of genetic mutations can initiate acute myeloid leukemia [1]. Several mutations associate with specific DNA methylation patterns [2]. The mechanisms how such aberrant methylation patterns are established remain enigmatic. PML-RARα is an oncogene that induces acute promyelocytic leukemia (APL). Recently, we dissected the methylome of APL at base pair resolution [3]. Genome-wide hypermethylation in CpG rich regions was a representative characteristic of APL. Intriguingly, PML-RARα binding-sites remained non-methylated and appeared protected from the actual hypermethylation phenotype in APL. In contrast to this protection from DNA methylation, PML-RARα target-genes are silenced via recruitment of histone deacetylases (HDACs) to PML-RARα binding-sites [4, 5]. Surprisingly, the actual binding of PML-RARα takes place at accessible chromatin [6], which together with our data underscores that heterochromatin formation at PML-RARα targeted genes is not required for APL pathogenesis. Interestingly, the repressive action of PML-RARα can be efficiently switched off upon ATRA treatment, which is directly translated to increased histone acetylation and gene expression without a change in DNA methylation [5]. While histone deacetylation is directly linked to HDAC recruitment by PML-RARα it remains unclear how specific DNA methylation patterns are being established. In addition, the fundamental question emerges, whether DNA methylation aberrations contribute to leukemia or remain downstream events in a PML-RARα transformed cell.

Recent whole genome sequencing data from human APL patients and a mouse APL model system consolidated that PML-RARα is the initiating event in APL, but underscored that cooperating mutations (i.e. mainly FLT3) are needed to induce the disease [1]. In this view, PML-RARα provides a competitive growth advantage for t(15;17)-positive cells and increases the probability for a cooperative mutation in a subsequent event. Similar to this model, we did not observe methylation changes in PML-RARα transduced cells, which otherwise already showed the characteristic differentiation block and enhanced proliferation. The same was observed in preleukemic PML-RARα mice, which did not yet show aberrant DNA methylation. These findings suggest that the APL methylation pattern is established at later stages of the disease, probably at the time when the co-operating mutation occurs. Notably, we found an increased variability in DNA methylation in APL, which supports the model that aberrant methylation is established at late stages of APL development. Potentially, co-operating mutations that drive clonal evolution in APL might be responsible for the methylation pattern seen rather than PML-RARα itself. Now, what is the significance of the observed methylation changes?

Hypermethylation in APL patient samples frequently took place at genomic regions regulated by the polycomb repressive complex 2 as determined by SUZ12 and REST binding-sites in embryonic stem cells (ES-cells). Moreover, we observed that more than half of the ES-cell SUZ12 binding-sites are covered by SUZ12 in APL-patient cells as well. Sites covered by SUZ12 in both ES-cells and APL patient sample remained unmethylated. Strikingly, sites, which were bound by SUZ12 in ES-cells but are devoid of SUZ12 in the APL-patient sample were frequently found to be hypermethylated. Again this hints towards a mechanism in the establishment of the APL specific DNA methylation patterns. DNA methylation spreads stochastically at regions which become accessible to DNA methyltransferases due to loss of protection by transcription factors. Initially, this model was reported based on data from mouse ES-cells [7]. Recently, similar conclusions were drawn from ENCODE data based on accessible chromatin, which was evaluated as Dnase I hypersensitive sites [8]. Notably, significant negative correlations were observed between transcription factor expression levels and binding site DNA methylation at cognate recognition sites within accessible chromatin. Taken together, this strongly supports the hypothesis that DNA methylation patterns arise from passive loss of transcription factor binding.

The multitude of DNA methylation patterns in the different leukemia subtypes might reflect the mode of action of its driver genes. Recently, the influence of IDH mutations and MLL oncogene induced leukemia has been analyzed at comparable resolution [9]. While MLL induced leukemia results in genome-wide hypomethylation, IDH mutations cause DNA hypermethylation. The MLL protein connects a histone methyltransferase, known to set activating histone marks, with an unmethylated DNA binding motif. Although there is a close link between MLL and its direct target genes in up-regulation, MLL induced hypomethylation is not preferentially associated with its target sites. Similar to this, hypermethylation associated with IDH mutation is not linked to targeting processes of the modified IDH enzyme itself. Hypermethylation basically results from blocking of DNA de-methylation processes, which is conducted by TET enzymes.

In summary, initiation of leukemogenesis by PML-RARα occurs independently from DNA methylation. Aberrant DNA methylation in APL rather evolves through co-operating mechanisms, e.g. loss of protective transcription factor binding, that occur during increased cycling in leukemogenesis.
